# Assessing a novel second generation laryngeal mask airway using the ‘ADEPT’ approach: results from the LMA® Protector™ observational study

**DOI:** 10.1007/s10877-022-00910-5

**Published:** 2022-09-05

**Authors:** A. Ní Eochagáin, V. Athanassoglou, A. Cumberworth, O. Morris, S. Corbett, H. Jefferson, E. P. O’Sullivan, J. J. Pandit

**Affiliations:** 1 Consultant Anaesthetist St. James’s Hospital, Dublin, Ireland; 2 Oxford University Hospitals NHS Foundation Trust, Oxford, UK; 3 University of Oxford, Oxford, UK; 4 St John’s College, Oxford, OX1 3JP UK

**Keywords:** Supraglottic airway, Airway management, Oxygenation, Laryngeal mask

## Abstract

To address the problem of lack of clinical evidence for airway devices introduced to the market, the Difficult Airway Society (UK) developed an approach (termed ADEPT; Airway Device Evaluation Project Team) to standardise the model for device evaluation. Under this framework we assessed th*e* LMA Protector, a second generation laryngeal mask airway. A total of 111 sequential adult patients were recruited and the LMA Protector inserted after induction of general anaesthesia. Effective insertion was confirmed by resistance to further distal movement, manual ventilation, and listening for gas leakage at the mouth. The breathing circuit was connected to the airway channel and airway patency confirmed with manual test ventilation at 20 cm H_2_0 (water) pressure for 3 s. Data was collected in relation to the time for placement, intraoperative performance and postoperative performance of the airway device. Additionally, investigators rated the ease of insertion and adequacy of lung ventilation on a 5-point scale. The median (interquartile range [range]) time taken to insertion of the device was 31 (26–40[14–780]) s with the ability to ventilate after device insertion 100 (95% CI 96.7- 100)%. Secondary endpoints included one or more manoeuvres 60.3 (95% CI 50.6—69.5)% cases requiring to assist insertion; a median ease of insertion score of 4 (2–5[3–5]), and a median adequacy of ventilation score of 5 (5–5[4–5]). However, the first time insertion rate failure was 9.9% (95% CI 5.1—17.0%). There were no episodes of patient harm recorded, particularly desaturation. The LMA Protector appears suitable for clinical use, but an accompanying article discusses our reflections on the ADEPT approach to studying airway devices from a strategic perspective.

## Introduction

Following the introduction of the Laryngeal Mask Airway (LMA) Classic™ (LMA™ North America, Inc., San Diego, CA, USA) in 1989 [1], the use of laryngeal mask airway devices in elective and emergency airway management has become widespread in clinical practice and is used in more than half of general anaesthetic operations in the UK [2, 3]. Although the original device is still in use, there have been several changes to the design which has led to the development ‘second generation’ supraglottic airway devices (SADs). These devices aim to overcome the reported complications associated with SAD use, including the risk of aspiration, potentially difficult placement, poor seal and sore throat postoperatively. Notably, though, these complications are argued to arise after blind insertion and alternative methods have been proposed [4]. The classification of SADs by ‘generation’ is discussed elsewhere [5–7].

This proliferation of SADs, coupled with a recognised lack of clinical evidence supporting use of the new devises before they were brought to market led the Difficult Airway Society (DAS) to develop guidance (termed ADEPT after the Airway Device Project Evaluation Team) as a framework under which clinical studies could be facilitated [8]. It was under this framework (described further in an accompanying article [9]) that we undertook a prospective observational cohort study of the LMA® Protector™ to investigate its clinical performance characteristics.

**Fig. 1:**
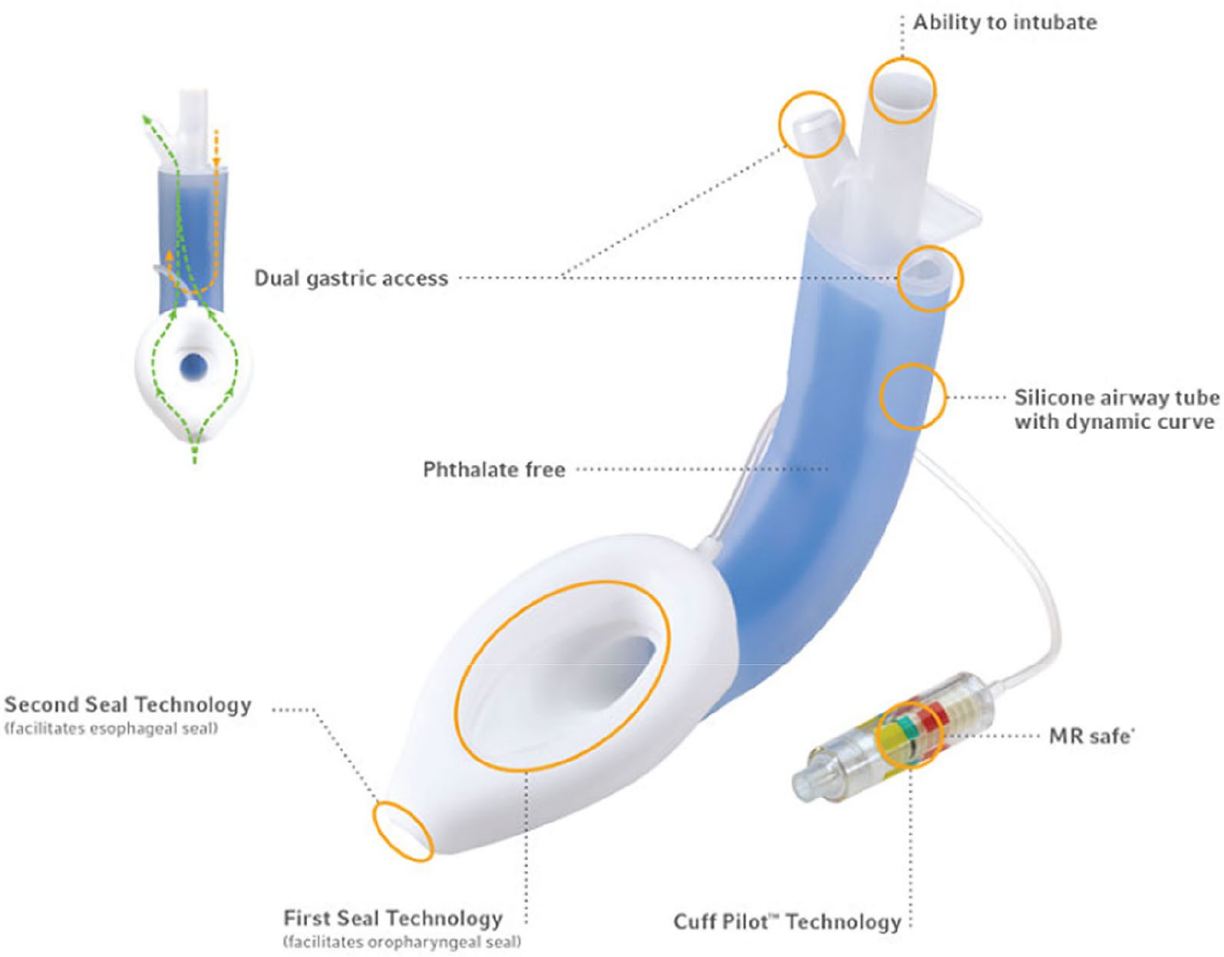
LMA® Protector™ (figure provided by courtesy of Teleflex Medical, Co. Westmeath, Ireland)

The LMA® Protector™ (Teleflex Medical, Co. Westmeath, Ireland) can be characterised by a taxonomy described elsewhere [10]. It is a single use device that incorporates a number of improvements which aim to reduce the already low complication rate associated with SAD use. The device is an evolution of previous second generation SADs developed by Teleflex which are in everyday use around the world (LMA® SupremeTM, LMA® ProsealTM, LMA® GuardianTM) which all have a good record of safety and effectiveness [11–15]. These evolutions include: (a) dual gastric channels which continue distally and enter a chamber located behind the cuff. The chamber further narrows distally into an orifice located at the end of the cuff to communicate distally with the upper oesophageal sphincter. A suction tube may be attached to the male drainage port around the laryngeal region or a well lubricated gastric tube may be passed through the female drainage port to the stomach (Fig. 1); (b) the airway tube and cuff are 100% silicone, phthalate free and designed to conform to the airway anatomy. Silicone cuffs have been shown to reduce risk of sore throat and achieve higher seal pressures compared with PVC cuffs. An integrated cuff pressure indicator for single-use airway management devices that enables continuous cuff pressure monitoring at a glance and facilitates easy, accurate adjustment when necessary; a bite block; a fixation system which prevents proximal displacement during use ensuring that the distal end seals around the upper oesophageal sphincter.

## Methods

This multicentre, international study took place in Great Britain and Ireland over five hospital sites. Ethics approval was granted by local ethics boards to undertake a prospective observational cohort study of the LMA® Protector™ in line with DAS ADEPT guidelines for device evaluation [8]. The study was conducted at St. James’s Hospital, Dublin (two hospitals) and Oxford University Hospitals NHS Foundation Trust between 2019–2021 (three hospitals) and there were no changes to methods after study commencement. The study was prospectively registered with clinicaltrials.gov (ClinicalTrials.gov Identifier: NCT03664700).

In order to ensure uniformity of insertion technique, participating anaesthetists were provided with didactic and practical instruction on the placement and correct use of the LMA® Protector™. A variety of manikin heads were used to afford anaesthetists the opportunity to practice insertion of the LMA® Protector™ and to practice placement of a nasogastric (NG) tube via its gastric port. Only the study investigators enrolled patients and placed the device during the study.

We enrolled male and female patients aged 18 years and older undergoing operation under general anaesthesia with a supraglottic airway device. Included patients were: adults > 18 yrs having a general anaesthetic; participants willing and able to give informed consent for participation in the study; ASA 1—3 category patients; patients clinically deemed suitable for a SAD insertion as based on patient and operation factors. Exclusion criteria were if patients refused consent; age < 18 years; patients requiring intubation for the operation such as risk of regurgitation; ASA 4 and above; predicted difficult tracheal intubation.

After signed informed consent, and arrival in the anaesthetic room standard monitors including peripheral capillary oxygen saturation (SpO2), electrocardiography, non-invasive blood pressure and capnography, were applied. Intravenous access was obtained and patients were positioned supine with their head placed on a pillow, with neck flexed and head extended, consistent with current practice for LMA insertion. Premedication (0.03 mg kg^−1^ midazolam, 1–2 μg kg^−1^ fentanyl) was permitted. Oxygen was administered via a face mask at 6–10 L/min for 3 min before general anaesthesia was induced using 2–3 mg kg^−1^ IV propofol. If neuromuscular blockade was administered the drug and dose was recorded and is presented in the results below, The LMA® Protector™ was inserted once an adequate depth of anaesthesia was achieved, using loss of verbal communication and eyelash reflex as a guide. General anaesthesia was maintained with sevoflurane in air/O_2_.

Insertion of the LMA followed a standardized procedure similar to the technique described for the LMA®Classic™ Airway [16]. The convex surface was lubricated with water-based gel before grasping the integral bite block and advancing it behind the tongue. Next, the LMA was guided from the hard to the soft palate, down the posterior pharyngeal wall, and into the hypopharynx where resistance is felt. Here it was seated with the airway channel cuffed around the laryngeal opening and the posterior part of the device with the distal gastric port directed at the upper oesophageal entrance. Effective insertion of the device was confirmed by resistance to further distal movement, manual ventilation resulting in effective chest wall movement, observing movement of the reservoir bag in response to spontaneous ventilation, and listening for gas leakage at the mouth [17, 18]. The breathing circuit was connected to the airway channel and airway patency confirmed with manual test ventilation at 20 cm H_2_0 (water) pressure for 3 s effected by use of the adjustable pressure-limiting valve on the anaesthetic machine.

Data was collected in relation to the placement, intraoperative performance and postoperative performance of the airway device. The schedule of observations is supplied in Table 1. The named investigators who have significant experience with research data collection, recorded primary and secondary outcome data as outlined above. Additionally, investigators rated the ease of insertion and adequacy of lung ventilation on a 5-point scale.

**Table 1:** Study Sequence

1. LMA protector (appropriate size) handed to anaesthetist
(a) Time starts after confirmation of adequate of anaesthesia post-induction
(b) LMA protector inserted
(c) LMA protector cuff inflated to top of green line (60cmH2O) on pilot cuff—note volume in cuff
(d) Anaesthetist allowed up to 3 insertions to secure what they clinically regard as best fit according to existing guidance [15]
(e)1st capnography trace obtained: time to insertion noted
If the anaesthetist not satisfied after × 3 attempts in total then time stops and data collection ends, and an alternative device, at the discretion of the anaesthetist, is used and noted
After successful placement:
(f) Measure leak pressure: set flow at 6L/min, increase APL valve pressure until audible leak starts; note at what level leak occurs
(g) Confirm correct placement of LMA Protector: perform suprasternal notch test and note result
(h) Pass nasogastric tube through gastric port, if clinically indicated
2. For ease of LMA insertion, score on 11-point numerical rating scale (0—very difficult to 10—very easy)
(a) Ease of insertion
(b) Quality of seal
(c) Protection from aspiration
3. Note type of anaesthetic used (e.g. volatile [which agent], TIVA and all drugs used)
4. During surgery note
(a) Loss of adequate airway ± Need to change airway
(b) Regurgitation
(c) Laryngeal spasm
5. Upon removal of device note
(a) Evidence of blood
(b) Evidence of aspiration
(c) Laryngeal spasm
6. Post operatively patient satisfaction measured
(a) Sore throat
(b) Pain on swallowing
(c) Oropharyngeal numbness
(d) Jaw pain
(e) Pain on speaking
Primary outcome measures
1. First go insertion success rate
2. First go successful ventilation rate
3. Percentage of complication free insertions
Secondary outcome measures
1. Time to first square capnography waveform
2. Lowest oxygen saturation level
3. Interventions needed to ensure airway patency
4. Quality of ventilation
(a) The quality of ventilation will depend on whether there was visible chest movement, tidal volume > 7 ml/kg, stable SpO_2_ and square capnography trace
5. Complication occurrence during insertion of device, during anaesthesia, and on device removal

The sample size was estimated as that which would be likely to yield at least one failure of insertion. Applying the analysis of binomial confidence intervals for observational studies, and assuming a first-time insertion failure rate of 5%, we estimated a sample size of 110 patients would be required to observe at least one first-time failure of insertion [19]. The final number of patients enrolled was therefore 111. The threshold of 5% does not indicate a clinically acceptable limit, but a likely rate based on preliminary experience of performance.

Data was analysed by GraphPad Prism version 8 (GraphPad, Salt Lake City, UT, USA). Data was recorded in Excel (Microsoft™) and imported into Prism v8 for analysis. All data was stored according to EU Directive 2019 on General Data Protection Regulations. Mean and standard deviation was used to describe normally distributed continuous variables; median and interquartile ranges (IQR) were used for non-normally distributed continuous variables. Categorical variables were expressed as number and percentage.

## Results

In total, 112 patients were enrolled from September 2019 to March 2021. One patient declined to participate in the post-operative questionnaire and so they were removed from the study and their results were not analysed. Table 2 outlines patient characteristics and Table 3 the type of surgery. Table 4 summarises the main results.

**Table 2:** Baseline characteristics of patients recruited (n = 111)

	Median (IQR [range]) or n, proportion (%)
Age (years)	68 (42–78[18–84])
Body weight (kg)	77 (66–83[36–107])
BMI (kg/m^2^)	25.5 (23.4–27.7[14.2–33.5])
Gender (male)	53, 47.8%
ASA class	ASA 1 47, 42.3%
	ASA 2 56, 50%
	ASA 3 8, 7.2%

**Table 3:** Details of Surgery

	Number, proportion (%); or Median (IQR[range])
Use of NMB	3 (2.7%)
Duration of Surgery (median (IQR[range])	80 (50-95 [20-180]) mins
Surgical Procedure; n,%	General Surgery 39, 35.1%
	• Mastectomy 18, 16.2% • EUA Perineal Abscess 6, 5.4% • I+D abscess 5, 4.5% • Inguinal Hernia repair 4, 3.6% • Fistulectomy 3, 2.7% • Microductectomy 1, 0.9% • Evacuation of breast haematoma 1, 0.9% • Pilonidal Sinus 1, 0.9%
	Orthopaedic; 30, 27% • Fusion of forefoot or ankle 8, 7.2% • THR/DHS 7, 6.3% • Removal of metal 6, 5.4% • Total Knee Replacement 3, 2.7% • External Fixation Ankle 2, 1.8% • Rotator Cuff Repair 2, 1.8% • ORIF elbow or radius 2, 1.8%
	Urology; 25, 22.5% • Ureteroscopy (+/− stent) 16, 14.4% • TURP/ TURBT 4, 3.6% • Cystoscopy 3, 2.7% • Repair of hydrocele 1, 0.9% • Bladder botox 1, 0.9%
	Plastic Surgery; 10, 9% • Plastic surgery of hand or forearm 6, 5.4% • EUA and botox 4, 3.6%
	Gynaecology; 7, 6.3% • Hysteroscopy 7, 6.3%

*NMB* neuromuscular blockade, *I* + *D* incision and drainage, *EUA* examination under anaesthetic, *TURP* transurethral prostatectomy, *TURBT* transurethral resection of bladder tumour, *THR* total hip replacement, *DHS* dynamic hip screw

**Table 4:** Main results

	Number, proportion %; or Median (IQR [range])
Insertion of LMA® protector™	1st attempt n, 91.9%
	2nd attempt n, 7.2%
	3rd attempt n, 0.9%
Time to insertion of LMA® Protector™	31 (26–40[14–780]s
Airway manipulations	1 or more airway manipulation required n, 60.3%
	Jaw thrust n, 74.8%
	Neck extension n, 67.5%
	Chin lift n, 6%
	Reposition device n, 4.5%
Ease of insertion rated by investigators	4 (2–5 [1–5])
Adequacy of ventilation rated by investigators	5 (5–5 [1–5])
Oropharyngeal leak pressure (mm Hg)	37 (30–40 [10–70])
Volume of air to achieve intra-cuff pressure of 60 cmH_2_O (ml)	14 (12–15 [10–40])
Ability to maintain good seal and adequate ventilation	110, 99.1%
Suprasternal notch test positive	96; 86.4%
NGT placement attempted	36; 32.4%
Major complications (desaturation to 91%)	1; 0.9%
Minor complications	39, 35%
	6, 5.4% immediately following device insertion
	1, 0.9% during maintenance of anaesthesia
	32, 28.8% upon extubation
Post-operative patient survey in PACU and at 24 h	Mucosal injury 19, 17.1%
	Mild post-operative sore throat 37, 33%
	Mild pain on swallowing 12, 10.8%
	Mild post-operative dysphonia 7, 6.3%
	Mild mouth pain 3, 2.7%
	Mild jaw pain 3, 2.7%
	Mild numbness of tongue 1, 0.9%

*PACU* Post anaesthesia care unit, *NGT* Nasogastric tube. There were no moderate or severe post-operative complications. There were no post-operative cases of vomiting, lip or tongue swelling, hearing changes, or ear pain. Ease of insertion score is out of 5. A good seal was regarded to be no audible leak and adequate tidal volumes (> 7 ml/kg, stable SpO2, square capnograph trace). Values are IQR(range)mean] or n, %

Insertion of the LMA® Protector™ was successful in 100 patients on first attempt (90.1%; in fact a failure rate twice as high as initially predicted), in 10 patients (9%) on second attempt and on the third attempt in one patient (0.9%). Complete failure to place the device did not occur in any patient. The median (IQR [range]) time taken to insertion of the device was 31 s (26–40[14–780)]. Airway manipulations were undertaken to place the device in 67 cases, 60.3(95% CI 50.6–69.5)% with neck extension (67.5%) and jaw thrust (74.8%) being the most commonly used manipulations.

The mean ease of insertion was rated by investigators as a median ease of insertion score 4 (2–5(3–5]), and a median adequacy of ventilation score of 5 [5–5[4, 5]). The LMA protector™ was found to provide a good seal with mean oropharyngeal leak pressure of 37 mmHg [(30–40[10–70] (IQR [range]). Overall, the LMA protector™ was able to maintain good seal and adequate ventilation in 110 patients 99 (95% CI 97.3–100)%; in one case the seal was deemed inadequate and so bag mask ventilation was instead performed successfully for the short duration of the case. For these purposes, a good seal was regarded to be no audible leak and adequate tidal volumes (> 7 ml/kg, stable SpO2, square capnograph trace). The median volume of air to achieve intra-cuff pressure of 60 cmH2O was 14 [12–15[0–40]) ml. The suprasternal notch test (tapping the suprasternal notch and observing simultaneous movement of a drop of lubricant at the proximal end of the drain tube) was positive in 96 patients, 86.4 (95% CI 80.12–92.84)%. Attempt at placement of a nasogastric tube was undertaken in 36 patients, 32(95% CI 23.7–41.1)% of cases and was possible on first attempt in all such cases.

Major complications were noted in one case where hiccoughing occurred immediately post induction followed by laryngospasm with desaturation to 91% for less than one minute. This resolved after the administration of 20 mg propofol and the case continued with the LMA Protector™, the patient was comfortable in recovery and no adverse sequelae were present. Minor complications were detected in 39 cases (35 (95% CI 26.2—40.0%); five of these cases (4.5 (95% CI 0.6–8%)) occurred immediately following device insertion (three cases of hiccoughing which spontaneously resolved and two cases of poor seal where the device was changed for a larger size. One complication occurred during the maintenance phase whereby the device required repositioning. Post-operative patient survey revealed that complications occurred in 55 cases (49.5(95% CI 40.2–58.8)%). Mucosal injury as evidenced by blood stains on the LMA Protector™ was documented in 19 patients (17(95% CI 10.1–24.1)%). Delayed patient reported complications included mild post-operative sore throat in 37 (33(95%CI 21.2–38.2)%), mild pain on swallowing in 12 (10.8(95% CI 5.0–16.5)%), mild post-operative dysphonia in 3 (2.7(95% CI 0.3–5.7)%), mild mouth pain in 1 (0.9(95% CI 0.8–2.6%), mild jaw pain in 1 (0.9(95% CI 0.8–2.6)%) and mild numbness of tongue in 1 (0.9(95% CI 0.8–2.6)%). No patient reported any moderate or severe post-operative complications. The degree of post-operative sore throat, dysphagia and dysphonia were described to be mild in nature and lasted for less than 24 h.

## Discussion

The main finding of this study is that the LMA Protector shows acceptable performance characteristics for clinical use, but success in different outcome domains need to be balanced in settling a niche for its use. Notably, the median insertion time was comparable to other devices like the Ambu®AuraGain™ and the LMA Supreme™ Second Seal™ [20], and ease of insertion and adequacy of ventilation via the device were both rated highly by investigators. However, the 10% failure rate on initial insertion exceeded our preliminary expectation of 5%, and also a suggested upper limit of 2.5% for supraglottic airway devices [19]. Recently Zaballos et al. reported an even higher first time failure rate of 16% in a larger trial of 280 patients [21].

Ours is not the first reported trial of the LMA Protector. Sng et al.’s description in 2017 [22] was in fact contemporaneous with the planning of our own trial, and the delays we then encountered are described in the accompanying article [9], and since then there exist over a dozen reports, including description of its use in different patient positions, emergency and elective settings, and for facilitating use of fibrescopy [13, 23, 24]. However, whereas most of these are individual case series or relatively small (but albeit appropriately statistically powered) randomised trials, ours is arguably the first study of a diverse patient cohort in real world settings. Our patient population was heterogenous in terms of gender, BMI, weight operative procedure and duration of surgery, which is more representative of standard practice than previous studies which focused on specific patient cohorts.

Perhaps for this reason, the number of complications noted in our study is higher than previous studies of the same device. This is unlikely to be due to misuse as all investigators were well experienced. Rather, it is a reflection of our study design which encouraged patient self-reporting of complications and which included a heterogenous case mix. Thus, our queries were framed as leading questions (e.g. ‘Do you have a sore throat?’), which may have prompted a higher positive response rate. Furthermore, this question was posed early on admission to the postoperative recovery unit, whereas other studies waited much longer to record this outcome, e.g. 18–24 h after operation [21–24]. Nevertheless our reported minor complication rate is not prohibitive to use of the device. The presence of blood on supraglottic airway devices upon removal is common [21–24]. Most studies report the occurrence of visible bloodstaining ranging between 0 and 50% [21–25]. The wide range in these figures may be attributed to slight differences in device size, material or shape and also due to differences in device insertion techniques affecting minor trauma to the oropharyngeal mucosa. Our data shows that traces of visible bloodstaining occurred in 19 (17%) of cases which is well within the normal range for similar SADs [26].

The advantages of a multi-centre design are always balanced against the heterogeneity of insertion techniques and experience of anaesthetist participants. Although registered as a clinical trial, it was a single-arm observational study that, if registered as an audit might have been expedited to completion. This delay became important as guidelines for airway management during the pandemic changed regularly and for a significant period the number of SADs used in our trial departments was reduced both due to a reduction in overall theatre workload and due to an increase in the use of tracheal tubes due to concerns regarding possible aerosolization of SARS-CoV-2 virus with the use of SADs [27, 28]. Our protocol permitted the use of neuromuscular blockade and active lung ventilation but in the event, only three such cases were included: the results are unchanged if these are excluded. Finally, our study was appropriately sized to describe performance characteristics, but it is important to appreciate that complications or adverse events with zero incidence still have an upper 95% CI of 3.3%, so we cannot exclude the possibility that these events not seen could still arise with a frequency of 3 in 100 [19]. However, we think that remains within acceptable performance limits.
